# The Cost Effectiveness of Coronary CT Angiography and the Effective Utilization of CT-Fractional Flow Reserve in the Diagnosis of Coronary Artery Disease

**DOI:** 10.3390/jcdd10010025

**Published:** 2023-01-07

**Authors:** Rex A. Burch, Taha A. Siddiqui, Leila C. Tou, Kiera B. Turner, Muhammad Umair

**Affiliations:** 1 Philadelphia College of Osteopathic Medicine, 625 Old Peachtree Rd NW, Suwanee, GA 30024, USA; 2 Charles E. Schmidt College of Medicine, Florida Atlantic University, 777 Glades Road BC-71, Boca Raton, FL 33431, USA; 3 The Russell H. Morgan Department of Radiology and Radiological Science, The Johns Hopkins Hospital, 601 N Caroline St, Baltimore, MD 21205, USA

**Keywords:** coronary artery disease (CAD), coronary computed tomography angiography (CCTA), cost-effectiveness analysis (CEA), cost analysis, ischemic heart disease, cardiovascular heart disease

## Abstract

Given the high global disease burden of coronary artery disease (CAD), a major problem facing healthcare economic policy is identifying the most cost-effective diagnostic strategy for patients with suspected CAD. The aim of this review is to assess the long-term cost-effectiveness of coronary computed tomography angiography (CCTA) when compared with other diagnostic modalities and to define the cost and effective diagnostic utilization of computed tomography-fractional flow reserve (CT-FFR). A search was conducted through the MEDLINE database using PubMed with 16 of 119 manuscripts fitting the inclusion and exclusion criteria for review. An analysis of the data included in this review suggests that CCTA is a cost-effective strategy for both low risk acute chest pain patients presenting to the emergency department (ED) and low-to-intermediate risk stable chest pain outpatients. For patients with intermediate-to-high risk, CT-FFR is superior to CCTA in identifying clinically significant stenosis. In low-to-intermediate risk patients, CCTA provides a cost-effective diagnostic strategy with the potential to reduce economic burden and improve long-term health outcomes. CT-FFR should be utilized in intermediate-to-high risk patients with stenosis of uncertain clinical significance. Long-term analysis of cost-effectiveness and diagnostic utility is needed to determine the optimal balance between the cost-effectiveness and diagnostic utility of CT-FFR.

## 1. Introduction

Coronary artery disease (CAD) is the leading cause of death in the United States, often associated with angina, stroke, and myocardial infarction [1,2]. In the setting of increasing morbidity and mortality attributed to this condition, CAD poses a significant economic burden to the healthcare system [3,4]. According to the American Heart Association (AHA), annual direct and indirect medical costs associated with CAD are estimated to be USD 89 billion and USD 99 billion, respectively [2]. On average, patients with CAD who have had major adverse cardiovascular events (MACE), as defined by myocardial infarction, stroke, or cardiovascular-related death, experience an additional USD 44,495 in 1-year healthcare costs when compared to patients without MACE [5].

The diagnosis of CAD is guided by several invasive and non-invasive imaging modalities that look for evidence of decreased myocardial perfusion, impaired myocardial contractility, and other anatomical changes. Coronary angiography (CAG) is considered the gold standard in the diagnosis of CAD, as it is both diagnostic and therapeutic [6]. However, CAG can be considered an unfavorable diagnostic option because this invasive procedure is associated with high costs and several periprocedural risks, including access site complications such as hematoma, hemorrhage, active extravasation, arteriovenous fistula, and pseudoaneurysm and catheter-related complications such as cholesterol embolism, local vascular injury, and dissection [7].

There are alternative non-invasive options that provide diagnostic and prognostic information to guide risk stratification, subsequent testing, and disease intervention [8]. Non-invasive tests may be classified as anatomical, which reveals pathologic changes to the arterial wall, or functional, which examines the consequences of these arterial changes, such as myocardial contractile dysfunction [8]. Anatomic tests include coronary artery calcium scoring (CACS) and coronary computed tomography angiography (CCTA) [8]. Magnetic resonance angiography (MRA) of coronary arteries is also a newer anatomic test under investigation with limited clinical use [9]. Functional tests include stress echocardiography, exercise electrocardiogram (EKG), Doppler ultrasound-derived flow reserve measurements, cardiac nuclear imaging with single-photon emission computed tomography (SPECT) or positron emission tomography (PET), and pharmacologic stress cardiac magnetic resonance imaging (MRI) [8].

CCTA has been recommended as a “gatekeeper” diagnostic test for invasive CAG [6,10]. It is assumed that patients with a high pretest probability of CAD will need invasive CAG, and that patients with a low-to-intermediate pretest probability do not require revascularization and can instead be screened by functional testing such as stress echocardiogram or nuclear medicine-based stress examinations [8,11,12]. Other noninvasive imaging modalities used for patients with a low-to-intermediate pretest probability of CAD are cardiac MRI and CCTA [13]. Ultimately, several factors including imaging cost, local availability of the test, and prevalence of disease influence clinical decision-making and determine the preferred test in the clinical setting [12,13].

In recent years, the Coronary Artery Disease Reporting and Data System (CAD-RADS) classification has been developed to standardize the reporting of CCTA findings in the evaluation of CAD and to facilitate the decision-making process surrounding further patient management [14,15]. The CAD-RADS classification score is determined by the degree of maximal stenosis found on anatomical CCTA, which provides standardized recommendations for further functional or invasive testing at higher degrees of stenosis [15]. 

The Prospective Multicenter Imaging Study for Evaluation of Chest Pain (PROMISE) trial revealed that CCTA used in conjunction with CAD-RADS classification can identify cases of prognostically significant lesions that would be otherwise missed by functional testing [16,17]. In light of the findings from the PROMISE trial and other clinical trials alike, the guidelines provided by the AHA, the Society of Cardiovascular Computed Tomography (SCCT) and the British National Institute for Health and Care Excellence (NICE) recommend CCTA to rule out obstructive CAD in low-to-intermediate risk patients with stable or acute chest pain [14]. 

Establishing the absence of CAD carries a high negative predictive value for MACE during index hospitalization and a low likelihood of such events in the next two years [18]. Alternatively, initial detection of CAD by CCTA may advise clinicians when to use invasive CAG appropriately. As a result, healthcare systems without a “gatekeeper” diagnostic test may experience higher costs of care compared to those with CCTA-based strategies. In addition to providing diagnostic evidence of obstructive CAD or normal coronary arteries, CCTA can detect non-obstructive CAD in approximately 40% of patients, which may guide atherosclerotic cardiovascular disease (ASCVD) risk-lowering initiatives, including lipid-lowering therapies that can lead to improved clinical outcomes [16].

A major question facing healthcare economic policy today is defining the role of CCTA in diagnosing CAD, especially in the light of national efforts to pursue more conservative diagnostic initiatives where “less is more” [19,20,21]. Conflicting evidence exists as to whether CCTA is indeed beneficial to patients; however, this may depend upon the specific indication, patient setting, suspected pretest probability, and the actual severity of CAD, among other factors [22]. Some randomized comparative effectiveness trials have demonstrated that early CCTA in emergency department (ED) triage for acute chest pain patients with suspected acute coronary syndrome (ACS) was associated with significantly decreased hospital admissions and reduced length of hospital stay compared to non-invasive methods [10,19]. Another study found that the use of CCTA for stable symptomatic outpatients without known CAD had no significant improvements in health outcomes [23]. They also suggested that CCTA may have caused more harm than benefit because of exposure to radiation, increased risk of nephrotoxicity, and adverse reactions from CCTA contrast materials [23]. In this regard, the present study aimed to investigate the diagnostic value of CCTA in the clinical setting by reviewing available data on the cost-effectiveness of CCTA compared to other CAD diagnostic strategies.

## 2. Materials and Methods

**Figure 1:**
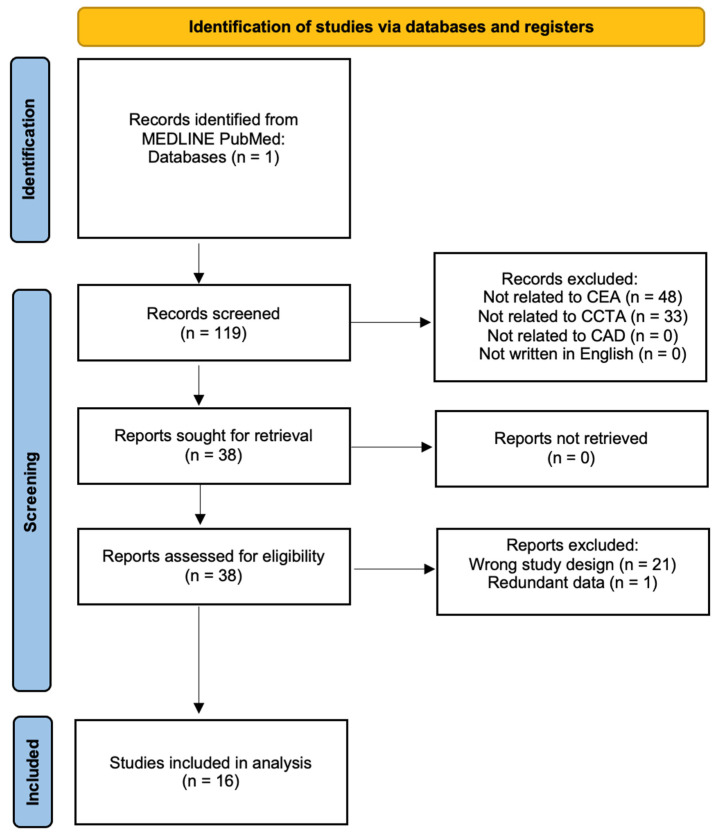
Flow diagram summarizing the identification of included studies.

A search was conducted through the MEDLINE database using PubMed. A total of 119 manuscripts were located in this database by using a combination of several key terms including “computed tomography,” “CT,” “coronary,” “angiography,” “CCTA,” and “cost-effectiveness.” Studies were selected if they conducted cost-effectiveness analysis (CEA) on the utility of CCTA in diagnosing CAD compared to other diagnostic modalities. The three inclusion criteria were determined as (1) the study must have employed their own data and/or data from a registry or healthcare institution to examine the cost-effectiveness of CCTA, (2) CAD must be the primary pathology being diagnosed by CCTA, and (3) all studies must be in English as demonstrated in the flow diagram (Figure 1) [24]. The manuscripts were screened by abstract and methodology to include only studies that fit the inclusion criteria. There were no restrictions on age, sex, or date of publication. Papers with redundant data, editorials, and other studies that qualitatively analyzed CCTA without exploring cost-effectiveness analysis were excluded. Manual forward and backward searches of citations and bibliographies of all relevant articles were performed. All searches were conducted in November 2021 and repeated in January 2022 by authors RB, LT, and TS. TS resolved any disputes regarding article selection. Ultimately, 16 manuscripts that provided the most relevant information were included in this literature review.

## 3. CCTA in Acute Chest Pain

In the U.S. alone, millions of patients present to the ED each year with complaints of acute chest pain, and many of these patients undergo unnecessary admission, additional testing, and inappropriate discharge, which leads to an increased economic burden on the healthcare system [23,25,26,27,28,29]. The utilization of CCTA has a high negative predictive value for CAD and has been shown to reduce hospital admission, length of hospital stay, and time to diagnosis, while also safely excluding acute coronary syndrome and preventing unnecessary CAG in acute chest pain patients (Table 1) [23,27,28,29]. Although the efficacy of CCTA is established, its long-term utility as a cost-effective test is still under consideration due to the lack of long-term cost-effectiveness studies and concerns regarding increased upfront costs as well as radiation exposure and risk of nephrotoxicity [21,22].

Multiple studies meeting the inclusion criteria for this review attempted to assess the long-term utility of CCTA when used in acute chest pain patients presenting to the ED. Goehler et al. (2020) utilized a Markov simulation model based on patients with low-intermediate risk of ACS to assess the long-term cost-effectiveness of CCTA relative to standard of care (SOC) and other management strategies. These strategies included SOC (serial cardiac biomarkers and functional testing), expedited ED discharge with close outpatient follow-up, and American College of Cardiology (ACC)/AHA guidelines [19]. Relative to other strategies, CCTA was associated with higher upfront costs (USD 4490 vs. USD 2513–USD 4144) and revascularization rates (5.2% vs. 2.6–3.7%), but over a lifetime, CCTA dominated SOC and ACC/AHA guidelines, and was cost-effective compared to expedited ED discharge (USD 49,428/quality-adjusted life year (QALY)). Additionally, CCTA led to decreased cardiovascular mortality (3-year: 1.04% vs. 1.10–1.17; 10-year: 5.06% vs. 5.21–5.36%) and remained cost-effective up to USD 90,000/QALY.

Ladapo et al. (2008) used a Monte-carlo microsimulation model to compare CCTA to SOC (serial cardiac biomarkers and functional testing) in low risk patients with acute chest pain and found that CCTA was cost-saving in women (decreased total cost USD 380) and associated with low incremental cost-effectiveness ratio (ICER) in men (USD 6400/QALY) [10]. This study concluded that CCTA was modestly more effective than SOC in both sexes (increased life expectancy by 8 days, increased QALYs 0.02). De Beule et al. (2010) applied a decision tree and mathematical model to assess the cost-effectiveness of CCTA in acute chest pain patients with initially negative troponins and no ST elevation on ECG [4]. Using two positivity thresholds for CCTA results (any degree of stenosis, or >50% stenosis in at least one vessel), abnormal CCTA findings guided the decision for hospital admission and subsequent testing. CCTA was found to be cost saving at both positivity thresholds for all pretest probabilities ≤71% with the most significant savings observed in the lowest pretest probability of 13%.

Taken together, these results suggest that while CCTA may cost more upfront, and lead to increased revascularization rates, it may improve outcomes and provide a more cost-effective approach over a lifetime in low-to-intermediate risk patients. When compared to SOC, this is likely due to the ability of CCTA to safely exclude ACS in the ED, and prevent unnecessary downstream admission and testing, while also decreasing the time to correct diagnosis and identifying appropriate candidates for invasive CAG (Table 1) [23,27,28,29].

**Table 1:** Summary of Major Cost Effectiveness Studies for CCTA in Patients with Acute Pain Syndromes.

Author	N	Study Population	Study Conclusion
Ladapo et al., 2008 [10]	N/A	Hypothetical patients with low-risk chest pain presenting to the ED	CCTA was cost-saving in women (decreased total cost USD 380) and associated with low incremental cost-effectiveness ratio (ICER) in men (USD 6400/QALY). This study concluded that CCTA was modestly more effective than SOC in both sexes (increased in QALYs 0.02).
Goehler et al., 2020 [19]	1000	Patients enrolled in the “Rule Out Myocardial Infarction/Ischemia Using Computer Assisted Tomography” (ROMICAT) II trial with low-intermediate risk of ACS who presented to the ED with suspicion of ACS	CCTA dominated SOC and ACC/AHA guidelines, and was cost-effective compared to expedited ED discharge (USD 49,428/QALY)
Hoffmann et al., 2012 [23]	1000	Multicenter trial with patients 40 to 74 years old with suspected ACS without ECG changes	In patients with suspected ACS, utilizing CCTA improved clinical decision making with no decrease in the total cost of care (USD 4289—CCTA group and USD 4060—standard of care, *p* = 0.65)
Litt et al., 2012 [28]	1370	Multicenter study with patents over 30 years old with low-to-intermediate risk of possible ACS	In comparison to the standard of care, patients in the CCTA group had reduced hospital length of stay and a higher detection of ACS
Goldstein et al., 2011 [29]	699	Multicenter randomized clinical trial across 16 EDs with randomized patient allocation suspected of ACS	Utilization of CCTA resulted in 54% reduction in time to diagnosis and 38% lower costs compared to perfusion testing (USD 2137—CCTA vs. USD 3458—MPI)

Abbreviations: ACS—Acute Coronary Syndrome, ECG—Electrocardiogram, CCTA—Coronary Computed Tomography Angiography, ED—Emergency Department, MPI—Myocardial Perfusion Imaging.

## 4. CCTA in Stable Chest Pain

For patients presenting with stable chest pain in the outpatient setting, functional testing with SPECT, stress echocardiography or exercise ECG has traditionally been used to diagnose CAD and act as a “gatekeeper” to invasive CAG. However, growing evidence suggests that in low-to-intermediate risk patients, CCTA may provide a more accurate and efficient diagnosis of CAD while improving health outcomes related to cardiovascular death and myocardial infarction (Table 2) [30,31,32]. Furthermore, there is evidence to suggest that the use of CCTA prior to CAG is cost saving [33,34]. However, the higher upfront costs, increased invasive testing, and increased revascularization related interventions associated with CCTA has led to uncertainty in utility when compared to functional testing. Thus, long-term cost-effectiveness data comparing CCTA to functional testing is needed to evaluate potential health benefits to patients over a lifetime.

Lee et al. (2015) performed a retrospective cohort study comparing CCTA to SPECT in 1632 patients with a pretest probability between 10 and 90% that presented to the outpatient clinic with chest pain [6]. At one year follow-up, CCTA was found to be marginally more effective (0.90824 QALY vs. 0.90603 QALY) and less expensive (USD 1760 vs. USD 2264) when compared to SPECT [6]. Genders et al. (2013) compared CCTA to exercise ECG by conducting a prospective cohort study of 471 outpatients that presented with stable chest pain [35]. A Markov simulation model was used to assess lifetime healthcare costs and QALYs. In patients with a pretest probability of less than 70%, CCTA was found to be less expensive and more effective over a lifetime when compared to exercise ECG in 100% of simulations (cost savings EUR 444, 0.0035 QALYs gained). At pretest probabilities greater than 70%, CCTA was cost-effective in 59% of simulations.

Dewey and Hamm (2007) used a hypothetical patient cohort and a decision tree model to compare the use of CCTA, coronary artery calcium score (CACS), or stress MRI to traditional tests including exercise ECG, stress echocardiography, and invasive CAG [36]. CCTA was found to be the most cost-effective test for patients with a pretest probability of CAD between 10 and 50% with costs ranging from EUR 4435 to EUR 1465 for correct diagnosis. CAG was the most cost-effective when the pretest probabilities reached 70%. Bertoldi et al. (2017) employed a decision-analytical model to compare functional and anatomical testing strategies in a hypothetical cohort with a pretest probability between 20 and 70% [37]. The exercise ECG strategy was found to be the least costly and least accurate, while the CCTA strategy was found to be the most cost-effective with stress echocardiography performing nearly as well (ICER USD 286 and USD 305, respectively).

Several studies included in this review also compared sequential diagnostic strategies involving CCTA or other modalities to evaluate patients with stable angina. Bertoldi et al. (2016) used a Markov simulation model to compare eleven different diagnostic strategies in a hypothetical patient cohort with pretest probabilities ranging from 20 to 70% [38]. The diagnostic strategies began with either exercise ECG, SPECT, stress ECHO, stress MRI, CCTA, or CAG, and then progressed to subsequent testing depending on results. The CCTA first strategy was the most cost-effective option amongst low-to-intermediate risk patients (0.3 QALYs gained, ICER USD 3,100). Min et al. (2010) investigated sequential diagnostic strategies involving CCTA or SPECT as the initial test by performing a decision analysis based on patients with pretest probabilities ranging from 10 to 80% [39]. Both strategies utilizing CCTA dominated all other strategies up to 80% CAD prevalence and yielded favorable ICERs (CCTA first USD 17,516, CCTA only USD 20,429).

**Table 2:** Summary of Major Cost Effectiveness Studies for CCTA in Patients with Stable Pain Syndromes.

Author	N	Study Population	Study Conclusion
Lee et al., 2015 [6]	1632	Retrospective cohort study in patients with a pretest probability between 10–90% for ACS	At a one-year follow-up, CCTA was more cost-effective in terms of life quality (0.00221 QALY) and cost (USD 513) compared to SPECT, although SPECT performed better than CCTA in patients with a pretest probability of 30–60%
Genders et al., 2013 [35]	471	Prospective cohort study in patients with stable chest pain utilizing a Markov simulation model	In patients with a pretest probability below 70%, CCTA was less expensive and more effective (cost savings EUR 444, 0.0035 QALYs gained) in 100% of simulations
Dewey and Hamm, 2007 [36]	N/A	Hypothetical patient cohort with a decision tree model	Utilization of CCTA was most cost-effective in patients with pretest probability between 10–50%, with CAG being most cost-effective with pretest probability above 70%
Bertoldi et al., 2017 [37]	N/A	Hypothetical patient cohort with a pretest probability between 20% and 70% for ACS	CCTA strategy was most cost-effective (cost-effectiveness ratio of USD 286 per correct diagnosis)
Bertoldi et al., 2016 [38]	N/A	Hypothetical patient cohort using the Markov simulation model	CCTA driven strategy was the most cost-effective among low-to-intermediate risk patients (0.3 QALYs gained, ICER USD 3100) *
Min et al., 2010 [39]	N/A	Patients with a pretest probability for ACS between 10–80%, base case being a 55–year-old male with a 30% risk of obstructive CAD	Strategies utilizing CCTA prevailed over other strategies with favorable ICERs (CCTA first USD 17,516, CCTA only USD 20,429) *

* Stepwise diagnostic strategies are proceeded by further imaging modalities if previous ones are inconclusive or positive. Abbreviations: ACS—Acute Coronary Syndromes, CCTA—Coronary Computed Tomography Angiography, QALY—Quality Adjusted Life Years, SPECT—Single-Photon Emission Computerized Tomography, CAG—Coronary Angiography, ICER—Incremental Cost-Effectiveness Ratio.

## 5. Diagnostic Accuracy

CCTA has also demonstrated great diagnostic accuracy in several of the aforementioned studies, albeit not as accurate as the gold standard of coronary angiography [40]. This is demonstrated by multi-center studies (where CCTA has lower accuracy) compared to single-center studies [40]. Due to the slightly inferior diagnostic properties of CCTA compared to angiography, it can be used to rule out CAD in patients with a low-to-intermediate pretest probability, with direct angiography in patients with high pretest probability. However, this is entirely dependent on the sensitivity of CCTA. The higher sensitivity of CCTA has shown greater diagnostic accuracy (90% sensitivity correlated with USD 13,000/QALY) in patients with low pretest probability, females, and advancing age [37,40].

## 6. Limitations of CCTA

There are a number of limitations to take into account when considering CCTA as a diagnostic strategy. First, although advancements in technology and image acquisition have led to a considerable reduction in CCTA-related radiation exposure over the last decade [41,42,43], increased radiation exposure must be acknowledged when choosing CCTA over functional tests with no risk of exposure. Second, CCTA evaluations can be negatively affected by artifact from coronary artery calcification and preexisting stents. Artifact related to coronary artery calcium is variable depending on the anatomic distribution of each patient, and studies have shown differences in the performance of CCTA when considering Agatston cutoff scores [27,44,45]. Furthermore, newer scanners have shown improved performance in evaluating in-stent restenosis (sensitivity 90%, specificity 91%) [46]. Lastly, not all luminal narrowing of coronary arteries leads to myocardial ischemia [47,48], and while CCTA can identify anatomic stenoses with a high degree of sensitivity, specificity is limited due to the anatomic nature of the analysis. More specifically, it lacks the ability to provide functional information related to impaired blood flow across stenotic lesions. This leads to concern that patients with angiographically intermediate stenoses may undergo unnecessary revascularization due to a lack of relevant hemodynamic information.

## 7. Computed Tomography-Fractional Flow Reserve (CT-FFR)

CT-FFR is a novel non-invasive testing technique that applies computational fluid dynamics to traditional CCTA in order to predict pressure gradients across stenotic vessels. This allows CT-FFR to bridge the gap between CCTA and functional testing by measuring the hemodynamic significance of coronary artery stenoses. Recent meta-analyses have shown that CT-FFR demonstrates a 2-fold increase in specificity for flow-restricting lesions and an improved diagnostic index overall [49,50,51]. Furthermore, CT-FFR has been shown to be a better predictor of long-term prognosis compared to CCTA [52], while also maintaining a higher diagnostic performance across artifacts related to calcium and motion [53]. However, although the data suggest that CT-FFR with CCTA is superior compared to CCTA alone, the increased complexity of calculating CT-FFR is time-consuming and incurs increased costs relative to CCTA [54]. For this reason, healthcare systems must consider the pretest probability of CAD to optimize the use of CT-FFR for patient populations that stand to benefit from functional analysis of stenoses with uncertain clinical significance.

A recent meta-analysis assessed the use of CCTA and CT-FFR in confirming or excluding ischemic CAD across different pretest probabilities [54]. CCTA and CT-FFR decreased post-test probability to less than 15% when pretest probability was less than 61.3% or 59.3%, respectively. Post-test probability could be increased to greater than 85% when pretest probability was greater than 74.9% for CCTA or 54.5% for CT-FFR. These results imply that while both tests display an excellent negative predictive value in low-to-intermediate risk patients, CT-FFR is superior in identifying clinically significant stenoses in intermediate-to-high risk patients. Considering the higher costs associated with CT-FFR, this suggests that CT-FFR should be utilized when CCTA detects stenoses of uncertain clinical significance in patients with intermediate-to-high risk of CAD. Though the advantages of CT-FFR have been demonstrated in guiding treatment of CAD, large randomized clinical trials and long-term cost-effectiveness studies across different pretest probabilities are still needed to validate reduction in economic burden and improved health outcomes associated with its use.

## 8. Dual-Energy Computed Tomography (DECT)

DECT is an innovation in computed tomography technology that provides several benefits to the investigation of CAD when compared to single-energy computed tomography [55,56]. DECT uses two X-ray photon energy spectra to acquire datasets for each energy level in a single image acquisition, which allows for improved imaging quality and material interrogation. DECT imaging datasets are then used to generate multiple image types that aid in coronary imaging by reducing bloom artifact, increasing luminal visualization in stents and calcified plaques, enhancing coronary plaque characterization, improving assessment of myocardial perfusion defects, and decreasing radiation dose [55]. In addition, static and dynamic myocardial perfusion imaging acquired with DECT can be used to directly assess the hemodynamic significance of coronary stenoses by calculating myocardial iodine concentrations distal to stenotic lesions [56,57]. Accordingly, myocardial perfusion imaging acquired with DECT, as opposed to single-energy computed tomography, is superior with regard to sensitivity and specificity, as well as positive and negative predictive value for detecting significant stenoses [58,59].

The investigation of CAD with DECT provides improvements to many limitations associated with single-energy computed tomography, while also providing the option for functional assessment of coronary stenoses. Moreover, emerging evidence suggests that dynamic myocardial perfusion imaging may be complementary to the use of CT-FFR in increasing specificity of CCTA for identifying functionally significant stenoses [60]. However, due to lack of large multicenter studies and cost-effectiveness data, future studies are needed to validate its widespread use and establish its long-term cost-effectiveness compared to traditional diagnostic modalities.

## 9. Artificial Intelligence (AI) and the Cost-Effectiveness of CCTA

Artificial Intelligence (AI) within the field of radiology has experienced extensive growth over the past decade, and with cardiac radiology experiencing sharp increases in imaging volume, AI applications have aimed to optimize workflow, increasing radiologists’ productivity while reducing cost and improving patient care [61]. Most AI applications still need clinical validation before being implemented into clinical practice; however, the American College of Radiology has already compiled numerous FDA-approved AI algorithms for radiological use [62]. Studies in cardiac radiology have shown the potential for AI applications to help improve efficiency, time to diagnosis, prognostic stratification, and identification of ischemic lesions [62]. 

With respect to anatomic testing, there are a number of machine learning applications that have shown potential to improve the diagnostic performance of CT-FFR [63,64,65], and CCTA-based prognostication [66,67,68]. While these AI applications are not readily being used in the clinical setting today, future integration of AI into the diagnosis and management of CAD seems inevitable. With improvements in diagnostic accuracy, risk stratification, and downstream medical management, integrating AI may further improve the long-term cost-effectiveness of CCTA and CT-FFR in the future.

## 10. Conclusions and Limitations

Data from the above studies suggest that CCTA may be a cost-effective diagnostic strategy when used alone for the initial evaluation of CAD, or as part of a sequential diagnostic strategy. In low-to-intermediate risk patients with stable chest pain, CCTA provides a diagnostic option with the potential to reduce economic burden and improve long-term health outcomes when compared to functional testing. At pretest probabilities higher than 70-80%, CAG remains the most cost-effective option. 

Given the limited data in the current literature, and due to increased cost and added time to diagnosis, CT-FFR should be utilized in intermediate-to-high risk patients with stenosis of uncertain clinical significance. Long-term analysis of cost-effectiveness and diagnostic utility is needed in the future to determine the optimal balance between the cost-effectiveness and diagnostic utility of this new modality.

While the above studies provide useful insight into the long-term benefits of anatomical testing compared to functional testing, there are several limitations to be considered. First, the simulation and decision-analytic models used in these studies rely on inherent assumptions that may not replicate real-life variability in model inputs or clinical decision-making. Therefore, conclusions being derived from these models must be considered in the context of their respective sensitivity analyses to account for the possible influence of model assumptions on results. Second, variations in healthcare costs limit the generalizability of these studies to the geographical region and healthcare system from which the patient population was obtained. Lastly, due to variability in study design, clinical practice, test availability, patient presentation, patient preference, available expertise of interpretation, and follow-up due to extracardiac findings [27,69,70], the widespread utility of CCTA as a first-line imaging modality is still lacking in the settings of ACS and stable chest pain in patients with low-to-intermediate pretest probability. Thus, large-scale, multi-institutional randomized clinical studies are still needed to generalize these findings.

Recently, Karády et al. (2020) performed an individual-based Markov microsimulation using patient data from the PROMISE trial to compare anatomic and functional testing in patients presenting with suspicion of obstructive CAD [71,72]. The model was based on 10,003 patients at 192 clinical sites with real-life variations in clinical care, patient characteristics, and cost [71]. The authors found that anatomic approaches led to a gain of 6 months of perfect health over a lifetime when compared to functional testing (25.16 QALYs vs. 24.68 QALYs). The results from this study suggest that anatomic strategies may provide a more cost-effective diagnostic option over a patient’s lifetime when compared to functional testing. Although prospective long-term randomized controlled trials are still needed in the future, these findings further support the roles of CCTA and CT-FFR as part of a cost-effective diagnostic strategy for CAD.

## Data Availability

Not applicable.
